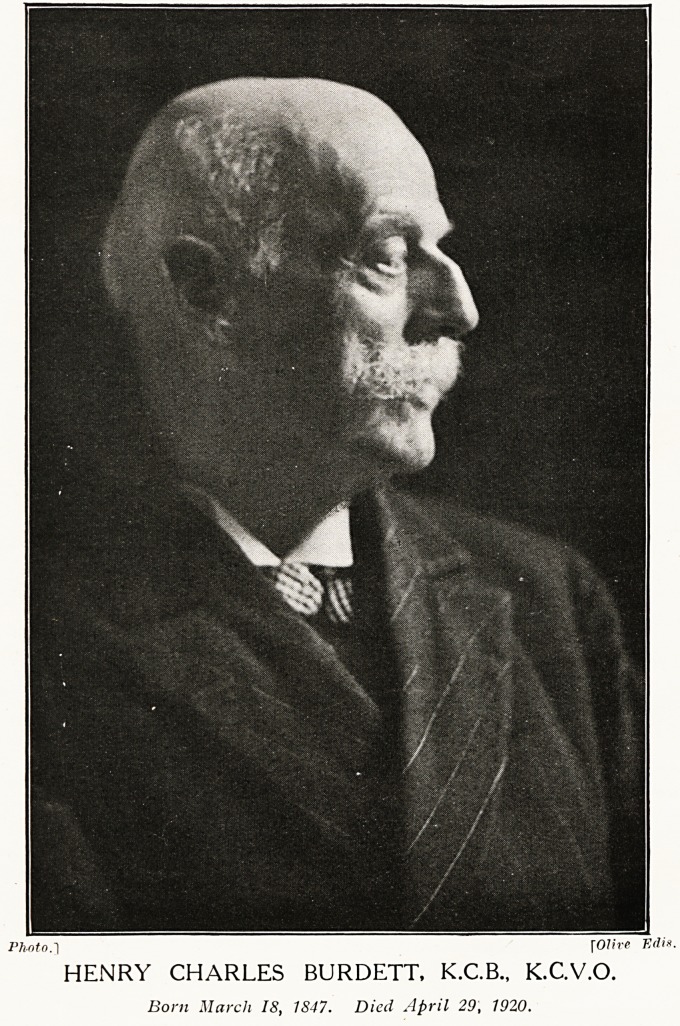# Death of Sir Henry Burdett

**Published:** 1920-05-08

**Authors:** 


### DEATH OF SIR HENRY BURDETT.

---

After an illness lasting several months,

SIR HENRY BURDETT, K.C.B., K.C.V.O.,

FOUNDER AND EDITOR

OF

**The Hospital**

AND

**The Nursing Mirror,**

Passed away on Thursday, April 29.

---

MARCH 18, 1847—APRIL 29, 1920.

**Requiescat in Pace.**

Photo.] [Olive Edis.

HENRY CHARLES BURDETT, K.C.B., K.C.V.O.

*Born March 18, 1847. Died April 29, 1920.*

## Figures and Tables

**Figure f1:**